# The Interactome of the VAP Family of Proteins: An Overview

**DOI:** 10.3390/cells10071780

**Published:** 2021-07-14

**Authors:** Christina James, Ralph H. Kehlenbach

**Affiliations:** Department of Molecular Biology, Faculty of Medicine, GZMB, Georg-August-University Göttingen, Humboldtallee 23, 37073 Göttingen, Germany

**Keywords:** VAPA, VAPB, MOSPD1, MOSPD2, MOSPD3, FFAT, interactome

## Abstract

Membrane contact sites (MCS) are sites of close apposition of two organelles that help in lipid transport and synthesis, calcium homeostasis and several other biological processes. The VAMP-associated proteins (VAPs) VAPA, VAPB, MOSPD2 and the recently described MOSPD1 and MOSPD3 are tether proteins of MCSs that are mainly found at the endoplasmic reticulum (ER). VAPs interact with various proteins with a motif called FFAT (two phenylalanines in an acidic tract), recruiting the associated organelle to the ER. In addition to the conventional FFAT motif, the recently described FFNT (two phenylalanines in a neutral tract) and phospho-FFAT motifs contribute to the interaction with VAPs. In this review, we summarize and compare the recent interactome studies described for VAPs, including in silico and proximity labeling methods. Collectively, the interaction repertoire of VAPs is very diverse and highlights the complexity of interactions mediated by the different FFAT motifs to the VAPs.

## 1. Introduction

The endoplasmic reticulum (ER) distributes widely throughout the cell and associates with many organelles including mitochondria, peroxisomes, endosomes and the plasma membrane. These associations are mediated by contacts termed membrane contact sites (MCS), which are regions of close apposition within 10–20 nm. They play key roles in lipid transport and synthesis, calcium homeostasis, organelle positioning and dynamics [1,2]. Several tethering proteins are involved in the formation of MCS by mediating protein–protein or protein–membrane interactions [3,4]. Deletion of tethering proteins reduces membrane contacts between organelles and affects physiological processes occurring at the MCS [4,5,6,7].

**Figure 1:**
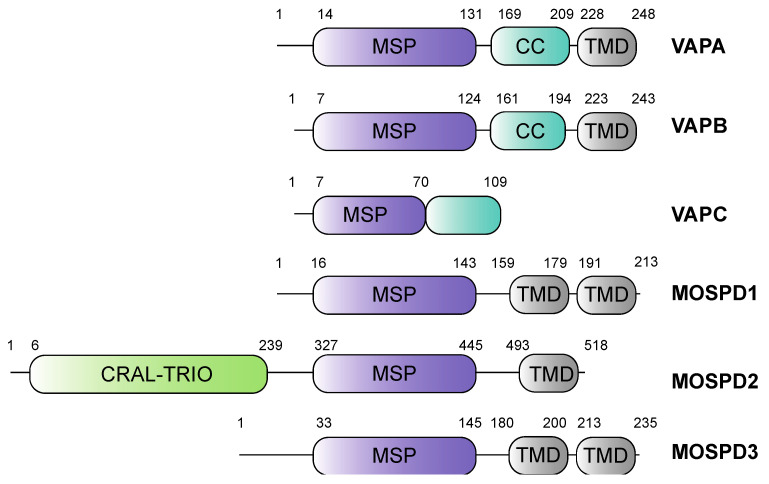
Domain structures of the VAP family of proteins. All VAP family of proteins have an MSP (Major Sperm Protein) domain. Both VAPA and VAPB contain a central coiled-coil domain (CC) and a C-terminal transmembrane domain (TMD). VAPC lacks the CC domain and the TMD. In addition to the MSP domain, MOSPD1 and MOSPD3 contain two TMDs at the C-terminal end, whereas MOSPD2 possesses a CRAL/TRIO (cellular retinaldehyde-binding protein and triple functional domain protein) domain at the N-terminal and a TMD at the C-terminal end.

Formation of MCS at the ER is mainly conferred by a family of proteins called VAMP-associated proteins (VAPs). A few examples of other tethers include ER-plasma membrane tethers (e.g., the calcium-dependent membrane tethers synaptotagmin 1, STIM1/Orai1 channels, anoctamin 8) [8,9], ER-mitochondria tethers (e.g., IP3R/VDAC, Fis1/BAP31) [10,11] and ER-endosome tethers (e.g., Protrudin/Rab7, ORP1L/ORP5) [12,13]. VAPs are highly conserved integral membrane proteins of the ER and ubiquitously expressed in eukaryotes [14,15,16,17]. The VAP family of proteins is characterized by the presence of a Major Sperm Protein (MSP) domain, with 22% sequence identity with the nematode major sperm proteins [18]. The MSP domain is characterized by seven immunoglobulin-like β-sheets and can form symmetric dimers [19]. The family includes the Vesicle-Associated-membrane Protein-associated proteins A and B (VAPA and VAPB), and the MOtile SPerm Domain-containing proteins 1, 2 and 3 (MOSPD1, MOSPD2 and MOSPD3) [20,21,22]. In humans, a sixth protein named Cilia and Flagella-Associated Protein 65 (CFAP65) also contains the MSP domain. It is expressed only in reproductive organs [23], whereas the other five are ubiquitously expressed [24]. MSP domains are present at the N-terminal region, as in the case of VAPA/B and MOSPD1/3, or more centrally, as in MOSPD2 [21] (Figure 1). This domain interacts with a short-conserved peptide motif named FFAT (two phenylalanines in an acidic tract) or variations thereof that are found in cytoplasmic regions in a wide variety of membrane-associated proteins localized in MCS between the ER and other organelles [20].

The interactome of the VAP family of proteins resulting from MSP–FFAT interactions has been studied in detail. In this review, we summarize the recent interactome studies performed on all the human VAP proteins and update the previous reviews on VAPs [18,25,26,27].

## 2. The Family of VAMP-Associated Proteins (VAPs) and VAP-Related Receptors

### 2.1. Vesicle-Associated-Membrane Protein Associated Proteins A and B

The first proteins of the VAP family were identified in *Aplysia californica* using a yeast two-hybrid screen [17]. VAP-33 (VAMP-associated protein of 33 kDa), a membrane protein required for neurotransmitter release, was shown to interact with VAMP (synaptobrevin). In humans, two proteins of the family have been described in detail: VAPA and VAPB. Both proteins are ER-integral transmembrane proteins with 63% sequence similarity. They consist of an N-terminal MSP domain, a central coiled-coil domain and a C-terminal transmembrane domain (Figure 1) [18]. As tail-anchored proteins, VAPA/B insert post-translationally into ER membranes [28,29]. VAPC is an alternatively spliced variant of VAPB containing the first 70 amino acids of its MSP domain and an additional unrelated 29 amino acids. VAPC lacks both the coiled-coil and the transmembrane domain (Figure 1) [14]. The MSP domain facilitates interaction with proteins carrying the FFAT motif localized in various organelles such as mitochondria, Golgi, peroxisomes, autophagosomes, endosomes and the plasma membrane [20,30,31,32,33,34,35,36,37,38,39,40,41]. A mutation in the MSP domain of VAPB (P56S) causes a familial form of amyotrophic lateral sclerosis (ALS) [42] and is also known to reduce FFAT motif-dependent interactions with some proteins [43]. VAPA/B undergo homo- or hetero-dimerization through their transmembrane domains and their MSP domains [14,22,44].

### 2.2. Motile Sperm Domain-Containing Protein 2

Loss of either VAPA or VAPB, or both, has no or only a minor effect on ER–organelle contacts [33,45,46]. This led to the notion that other tethering proteins might exist at MCSs that could recruit FFAT motif-containing proteins to the ER. A proteomic approach recently identified one such protein named MOSPD2 [21]. MOSPD2 is a single-pass membrane protein localized in the ER. It contains the signature MSP domain and thus belongs to the VAP-related proteins. It also contains an N-terminal CRAL/TRIO (cellular retinaldehyde-binding protein and triple functional domain protein) domain involved in lipid transport and a C-terminal transmembrane domain [21] (Figure 1). In contrast to VAPA and VAPB, which are present in animals, fungi and plants, MOSPD2 is only found in animals [21]. MOSPD2 mediates the formation of contact sites between the ER and endosomes, mitochondria and Golgi through its interaction with the FFAT motif containing proteins [21].

### 2.3. Motile Sperm Domain-Containing Proteins 1 and 3

In humans, there are two other proteins belonging to the MSP domain-containing proteins, namely MOSPD1 and MOSPD3. MOSPD1, identified for its role in mesenchymal differentiation [47], is an ER protein with an N-terminal MSP domain followed by two transmembrane domains (Figure 1). MOSPD3 is also an ER-localized MSP domain-containing transmembrane protein that is structurally similar to MOSPD1 and plays a role in right ventricle development and nuclear envelope reformation [22]. Recently, MOSPD1 and MOSPD3 were characterized as MCS proteins that interact with proteins carrying unconventional FFAT motifs [22].

## 3. FFAT Motifs

### 3.1. Conventional FFAT

**Figure 2:**
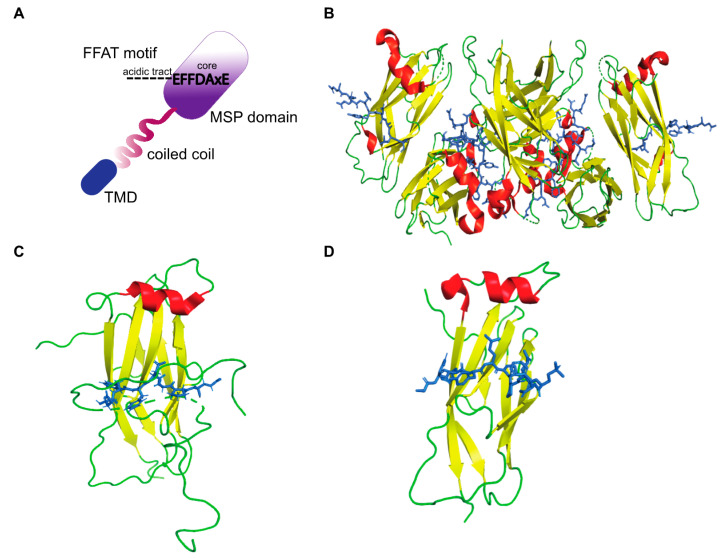
The structure of VAP-FFAT complexes. (**A**) Schematic representation of a conventional FFAT motif binding the MSP domain of VAP. The electropositive surface of the MSP domain binds the core residues of the FFAT motif, and the dotted lines represent the acidic tract. (**B**) The crystal structure of rat MSP-VAPA in complex with the ORP1 FFAT motif as described by Kaiser et al. [19] (PDB ID: 1Z9O). A tetramer formed by MSP-FFAT complex is shown. (**B**–**D**) The MSP domain is represented as ribbons (red: helix, yellow: β-sheet, green: loop), and the FFAT motif is represented as sticks (blue) that bind across the MSP domain. (**C**) The crystal structure of human MSP-VAPA in complex with OSBP-FFAT as described by Furuita et al. [51] (PDB ID: 2RR3). (**D**) Structure of human-MSP-VAPA in complex with STARD3 FFAT as described by Di Mattia et al. [52] (PDB ID: 6TQR).

The FFAT motif is a short linear motif responsible for targeting proteins to the ER [48] and also to the nuclear membrane [49]. Approximately 0.8% of all eukaryotic proteins contain FFAT motifs [50], including several human lipid binding proteins. A conventional FFAT motif consists of seven core residues, EFFDAxE (x is any amino acid) with an upstream acidic flanking sequence (Figure 2A). Two of the core residues bind to pockets within the MSP domain [19], and upstream flanking residues exert additional electrostatic interactions [51]. Structural analyses of VAP–FFAT complexes to identify mutations that specifically abolish the interaction of VAPA or VAPB with FFAT motifs showed that residues K94 and M96 of VAPA and K87 and M89 of VAPB are important for FFAT binding [19,20]. The crystal structures of MSP-VAPA/FFAT complexes show that two FFAT motifs bind to two VAPA-MSP domains [19]. The structure of the rat MSP-VAPA/ORP1-FFAT complex (PDB ID: 1Z9O) [19] confirmed that the FFAT motif binds a highly conserved, positive charged surface on the VAPA protein (Figure 2B). The FFAT residues exert hydrophobic van der Waals bonding with aliphatic residues from the MSP domain of VAPA and interact via intermolecular hydrogen bonds [19]. The crystal structures of human MSP-VAPA (PDB IDs: 2RR3 and 6TQR, Figure 2C,D) and rat MSP-VAPA were highly similar [51,52,53]. All the residues involved in binding of rat VAPA to the FFAT of ORP1 are also conserved in human VAPA-MSP [53]. The interaction is stabilized by electrostatic, hydrophobic and hydrogen bonding. In the case of human MSP-VAPA/STARD3-FFAT, the first few residues of the core FFAT sequence of STARD3 bound in a similar fashion to the ORP1 FFAT motif [52]. The difference in binding between STARD3-FFAT and ORP1-FFAT occurs in the second half of the core FFAT sequence. These VAP-FFAT structures were instrumental for further analysis of other VAP-FFAT/FFAT-like interactions [52].

### 3.2. Phospho-FFAT Motifs

A majority of VAP interactors do not have a conventional FFAT motif [25]. Di Mattia and colleagues [52] recently described a non-conventional FFAT motif where the acidic fourth residue (either aspartic acid or glutamic acid) in the consensus FFAT sequence is replaced by either serine or threonine. When their hydroxyl groups are phosphorylated, they gain a negative charge, facilitating binding to MSP domains. Phosphorylation of these motifs is essential for interaction with VAPs. Accordingly, they were named phospho-FFATs. Six of the previously identified VAP interactors were confirmed to have such a motif, namely STARD3, FIP200, MIGA2, PTPIP51, Kv2.1 and Kv2.2.

An unbiased screen for phospho-FFAT using an FFAT search algorithm in the human proteome identified 2079 proteins [52]. Among the known VAP interactors, 110 proteins were identified. It is important to note that the phospho-FFAT sequence has to be present in the cytoplasmic, unstructured part of the protein to be functional. About half of the identified proteins had both conventional and phospho-FFAT motifs. The authors also analyzed the structure of a complex of phospho-FFAT bound to VAPA or MOSPD2. The binding modes for both conventional and phospho-FFAT vary, especially in the second half of the core sequence. The distinction mainly arises from the geometry, as phosphoserine in the fourth position of the FFAT core is larger than aspartic acid, which might affect several contacts at MSP–FFAT interfaces. The authors postulate that phosphorylation acts as a switch for the interaction between VAPA/VAPB/MOSPD2 and phospho-FFAT motif containing proteins, thus controlling inter-organelle contact formation.

### 3.3. FFAT-Related FFNT Motifs

The majority of conventional-FFAT motifs have an acidic tract adjacent to the N-terminal side of the core region. One prominent exception is the FFAT motif of STARD3, where the acidic tract does not precede but follows the two phenylalanines [37]. Cabukusta and colleagues [22] described an FFAT-related motif, FFNT (two phenylalanines in a neutral tract), which lacks the residues corresponding to the acidic tract but contain neutral residues instead. FFNT motifs are preferred by MSP domains of MOSPD1 and MOSPD3. In FFNT motifs, the upstream acidic flanking residues are poorly conserved. The group described VAPA, VAPB and MOSPD2 as components of an ER-resident protein complex interacting with FFAT motifs or phospho-FFATs (in the case of MOSPD2). A second type of complex, on the other hand, contains MOSPD1 and MOSPD3 and rather interacts with FFNT motifs. Thus, two types of tethering complexes exist at the ER that interact with different FFAT motifs and form MCSs between ER and other organelles. A search for FFNT motifs in the human proteome and scoring the hits based on a position weight matrix identified CEP85, ANKLE2, ENTR1 and the inner nuclear membrane (INM) protein emerin as interactors of MOSPD1 and MOSPD3. The identification of both emerin and ANKLE2, both belonging to LEM domain proteins (Lap2, emerin and MAN1-LEM) of the INM, led the authors to speculate about the involvement of MOSPD3 in nuclear envelope dynamics during mitosis. Indeed, the authors showed that MOSPD3 is involved in the timing of post-mitotic nuclear envelope reassembly [22].

### 3.4. FFAT-Like Motifs

In light of conventional FFAT-, phospho-FFAT- and FFNT-motifs, the existence of additional variants appeared likely. Short linear motif discovery tools have predicted several FFAT-like motifs [54]. They were predicted based on certain guidelines: (1) the sequences of all conventional FFAT motifs were used; (2) they should be conserved across species; (3) they must have a cytoplasmic localization; (4) they should occur in an unstructured part of the protein [25,50,55]. A position weight matrix was used attributing an FFAT score of zero to the conventional FFAT [25]. A cut off score below 3 was used to define strong FFAT-like motifs. The predicted FFAT-like motifs using this search algorithm accounted for 50% of the total VAP interactors [25]. The remaining 50% of interactions could be mediated by other domains, e.g., the transmembrane domain of VAPs. Alternatively, more variants of FFAT-motifs are yet to be described.

## 4. Interactome of the VAP Family of Proteins

### 4.1. Approaches to Study the Interactome

**Figure 3:**
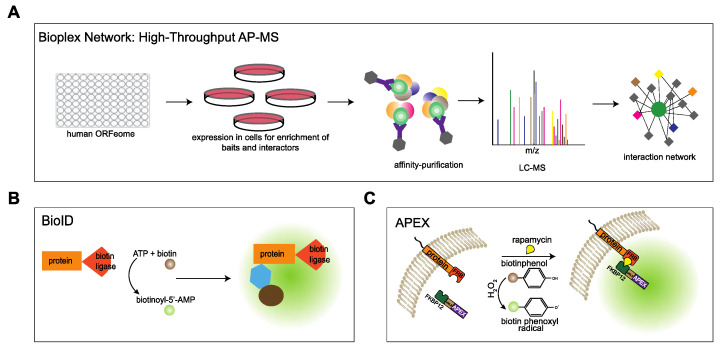
Approaches used to analyze the interactome of the VAPs. (**A**) Schematic of high-throughput interaction mapping using affinity-purification mass spectrometry. From the human ORFeome, a lentiviral library was created. The baits were expressed in HEK293T cells, subjected to affinity purification, and protein complexes were analyzed by LC-MS. All the interactors and baits were combined to generate a network of the human interactome [43]. (**B**) In a BioID approach, the protein of interest is tagged with biotin ligase and expressed in cells. The enzyme converts free biotin into reactive biotinoyl-5′-AMP, which reacts with primary amines of proximal proteins. The biotinylated proteins are enriched and identified using mass spectrometry [59]. (**C**) In APEX-based proximity labelling, the protein of interest is fused to ascorbate peroxidase (APEX). In the presence of H_2_O_2_, APEX converts biotin phenol into biotin phenoxyl radicals, which covalently label proteins in close proximity. In RAPIDS, the specific subcellular interactome of VAPB was analyzed by using rapamycin induced dimerization of FKBP12- and FRB-containing fusion proteins, followed by APEX2-dependent biotinylation of proteins and their identification by MS [60]. In this approach, SILAC allows a direct comparison between plus- and minus-rapamycin conditions and, thus, a very efficient elimination of the background.

**Figure 4:**
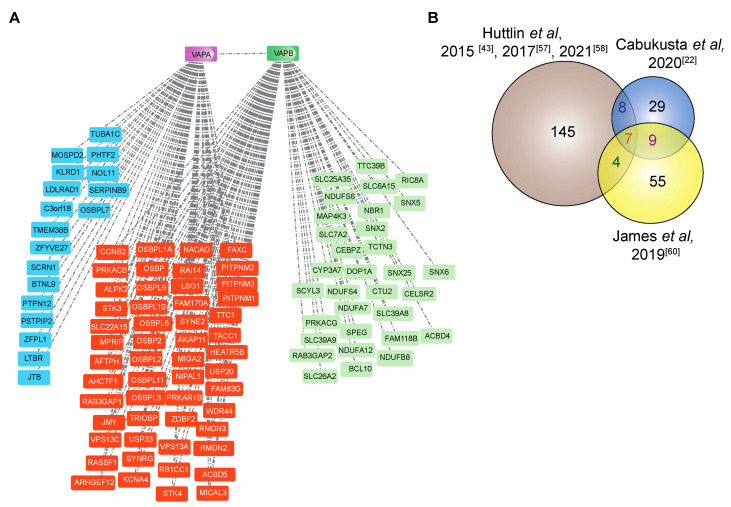
The interactome of VAPA and VAPB. (**A**) The Bioplex interaction network of both VAPA and VAPB in HEK293T cells reported by Huttlin et al. [43,57,58]. The proteins represented in blue are sole VAPA interactors, and those represented in green are sole VAPB interactors. The proteins represented in orange are common interacting partners of both VAPA and VAPB. (**B**) The Venn diagram shows VAPB interactors identified by Huttlin et al. (affinity purification-mass spectrometry) [43,57,58], by Cabukusta et al. (BioID followed by mass spectrometry identification) [22] and by James et al. (RAPIDS (rapamycin and apex dependent identification of proteins by SILAC) followed by mass spectrometry identification) [60]. See Table 1 for proteins that were identified in at least two studies.

The VAP proteins are major platforms for interaction of proteins with the ER. Different approaches have been used to investigate the interactome of the VAP family. In comprehensive studies to analyze the entire interactome of mammalian cells, Huttlin and coworkers [43] used a high-throughput affinity-purification mass spectrometry approach (Figure 3A) to identify interacting partners of 2597 human proteins in HEK293T cells. The authors used lentiviral expression of genes derived from the human ORFeome collection v. 8.1 in HEK293T cells [56] and affinity purification of C-terminally FLAG-HA-tagged baits and interactors followed by mass spectrometry. The resulting Bioplex (Biophysical Interactions of ORFeome-derived complexes) 1 network consisted of more than 20,000 interactions involving 7668 proteins. The network was further enhanced in Bioplex 2 [57] and recently in Bioplex 3 [58]. Mining the Bioplex network to search for the interactome of the VAP family of proteins, especially VAPA and VAPB, resulted in many known interaction partners of VAPA/VAPB in addition to new partners (Figure 4A).

In an approach that focused on specific proteins, Cabukusta and colleagues [22] used BioID to identify proteins interacting with all the VAP family of proteins. This technique is based on proximity-dependent biotinylation of proteins by a biotin ligase that is fused to a protein of interest. In the presence of biotin and ATP, the enzyme generates biotinoyl-5′-AMP that reacts with lysine residues of proximal proteins [59] (Figure 3B). Using BioID, unique and shared interaction partners of VAPA, VAPB, MOSPD1, MOSPD2 and MOSPD3 were identified. Additionally, MOSPD1 and MOSPD3 were shown to bind to unconventional FFAT-related motif containing proteins.

In an alternative approach that also monitors proteins in close proximity to a protein of interest, we used RAPIDS (rapamycin and apex dependent identification of proteins by SILAC) to analyze the interactome of VAPB [60]. This method combines ascorbate peroxidase (APEX2)-dependent biotinylation [61,62] and rapamycin-dependent targeting [63] of APEX to the protein of interest, followed by quantitative mass spectrometry using SILAC (stable isotope labelling with amino acids in cell culture) [60,64] (Figure 3C). RAPIDS allows a tight control over the subcellular localization of the enzyme (APEX2). In one approach, APEX2 was fused to the rapamycin interaction domain FKBP12, a GFP-tag and a nuclear localization signal (NLS) to target the protein to the nucleus. On the other hand, a tagged version of the protein of interest (e.g., HA-VAPB) was fused to an FRB (FKBP12-rapamycin binding) domain. Addition of rapamycin to transfected cells then led to dimerization of the two fusion proteins via their FKBP12- and FRB-domains, respectively. As a result, biotinylation of *nuclear* proteins that are in close proximity to VAPB occurred, allowing their subsequent enrichment and identification. To identify cytoplasmic interactors, APEX-FKBP12-GFP fusion protein lacking the NLS and an mCherry tagged FRB-VAPB was used [60].

### 4.2. The Interactome of VAPA and VAPB

More than 250 proteins are reported to interact with VAPA and/or VAPB (Figure 4) [22,43,57,58,60]. Exploring their interactome within the Bioplex network (Figure 4A) [43,57,58] shows that they share 50% of interacting proteins, the majority of which have FFAT motifs and are involved in lipid transfer between organelles. For instance, lipid transfer at ER-trans Golgi contact sites is mediated by interaction of FFAT domains of oxysterol binding proteins (OSBPs)/OSBP related proteins (ORPs) and VAPB [65,66,67,68]. OSBPs also target the trans Golgi network by another domain called PI4P-binding pleckstrin homology (PH) domain [69]. Another family of lipid binding proteins, NIR1, NIR2 and NIR3 (PITPNM 1–3) interact through their FFAT motifs with VAPB, and the interactions with NIR2 and NIR3 affect the structural integrity of the ER [31].

In addition to lipid transfer proteins, several other cytoplasmic proteins also bind to both VAPA and VAPB. The family of acyl-CoA binding domain proteins, ACBD4 and ACBD5 interact with VAPB to promote ER–peroxisome interactions [35,36], whereas VAPB interacts with the outer mitochondrial membrane protein PTPIP51 (RMDN3) to mediate calcium homeostasis [34]. Interestingly, the portion of PTPIP51 that binds VAPB has two FFAT motifs, one of which is highly conserved across all RMDN orthologues [55].

A ceramide transfer protein, CERT (STARD11), which transports ceramide between the ER and the Golgi, also contains dual targeting determinants (FFAT motif and PH domain) [67,70,71]. Furthermore, the late-endosomal sterol transfer proteins STARD3 and STARD3 N-terminal like (STARD3NL) interact with VAPA and VAPB via their FFAT motifs [37]. Both VAPA and VAPB interact with the autophagy proteins FIP200 and ULK1 through their FFAT motifs to modulate autophagosome biogenesis [39]. For several proteins, the interaction between VAPA or VAPB with its partners has been shown to be independent of FFAT motifs. YIF1A, for example, a component of the early secretory pathway that is important for membrane trafficking in dendrites, interacts with VAPB via its transmembrane regions [72].

Whereas most of the proteins that interact with VAPA and VAPB are located on organelles that are in contact with the ER, some proteins are found at other sites, for example at the inner nuclear membrane, nucleoli and eisosomes [49,50,59,73,74]. By mapping the interactome of a specific subcellular pool of VAPB localized particularly at the inner nuclear membrane (INM), several proteins of the INM were identified including emerin, TMEM43 and ELYS [60]. VAPB has been suggested to play a role in the nuclear transport of emerin [75]. The functional significance of other interactions, however, remains to be investigated. The VAPB interacting proteins identified in three different studies [22,43,60] are compared in Figure 4B.

The number of proteins that overlap between the three studies is rather small (seven proteins identified in all studies). The discrepancy could be explained by the different experimental setups used in each study. While in affinity purification methods [43,57,58] a careful choice of the lysis buffer is necessary to study stable interactions, proximity-based methods have their merits of enriching complexes that are not stable enough in harsh lysis buffer conditions [22,60]. Proteins found in at least two studies are listed in Table 1.

**Table 1:** Common interactors of VAPB as identified in three different studies.

Protein	Localization	Function	References
OSBP	ER-Golgi intermediate compartment	lipid transport	[32,65]
OSBPL9	ER-Golgi intermediate compartment	lipid transport	[32,65,66]
OSBPL11	ER-Golgi intermediate compartment	lipid transport	[32,65,66]
LSG1	nucleus	ribosome biogenesis	[43]
VPS13A	mitochondria	lipid transport	[76]
RMDN3	mitochondria	signaling	[33,34,60]
WDR44	endosome	-	[60,77]
RAB3GAP1	ER	GTPase activator, GTPase binding	[55,77]
RAB3GAP2	ER	GTPase activator, GTPase binding	[77]
OSBPL3	ER-Golgi intermediate compartment	lipid transport	[41]
OSBPL6	ER-Golgi intermediate compartment	lipid transport	[66]
OSBPL2	ER-Golgi intermediate compartment	lipid transport	[78]
VAPA	ER	lipid transport, membrane trafficking, calcium signaling	[39,55,76]
TTC1	cytoplasm	-	[25]
STK3	-	-	[25]
OSBPL10	ER-Golgi intermediate compartment	lipid transport	[25,60,78]
ACBD5	peroxisome	lipid transport	[35,36,60]
AHCTF1	nucleus, nuclear envelope	nuclear pore complex biogenesis	[25,60,79]
SYNE2	nuclear envelope	actin binding	[80]
emerin	nuclear envelope	-	[22,60,81]
OSBPL8	ER-Golgi intermediate compartment	lipid transport	[66]
RTN4	ER tubules	Formation and stabilization of ER tubules	[82]
ACSL3	mitochondria	lipid metabolism	[83]
CLCC1	ER, mitochondria associated membrane	-	[84,85]
PTP1B	ER	phosphotyrosine signaling	[86,87]
DDRGK1	ER	-	[85]
TMPO	nuclear envelope	lamin binding	[64,81,88]
ESYT1	ER	lipid transport	[4]

The proteins identified in all the three studies are represented in orange. Proteins overlapping in Huttlin et al. [43,57,58] and Cabukusta et al. [22] are in blue; in Huttlin et al. [43,57,58] and James et al. [60] in green; and in Cabukusta et al. [22] and James et al. [60] in magenta. Note that several established interaction partners of VAPB, such as CERT and STARD3, do not appear in any of the overlapping sections.

### 4.3. Interactome of MOSPD2

**Figure 5:**
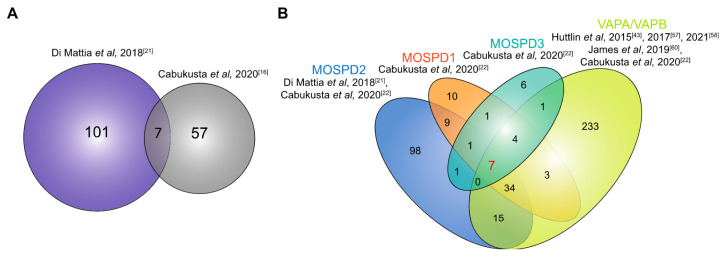
Interactome of the MOSPD proteins. (**A**). The overlap of proteins interacting with MOSPD2 as identified by Di Mattia et al. [21] and Cabukusta et al. [22]. The overlapping proteins are RAB3GAP1/2, CCDC47, RMDN3, ACSL3, FAF2 and TMPO. (**B**) Venn diagram showing the overlap of interacting partners of all five proteins of the VAP family. The proteins interacting with MOSPD2, MOSPD1, MOSPD3 and/or VAPA/VAPB were identified by Di Mattia et al. [21], Cabukusta et al. [22], Huttlin et al. [43,57,58], James et al. [60], as indicated. The seven proteins shown to interact with all proteins are TACC1, FNDC3A, ESYT1, ESYT2, ANKLE2, ZC3HAV1 and EIF2AK3.

A search for other tethering complexes that form MCSs between the ER and other organelles led to the identification of MOSPD2 as an FFAT-motif interacting protein [21,22]. As for VAPA and VAPB, an MSP domain in MOSPD2 enables it to bind to FFAT-containing proteins. Affinity purification-based mass spectrometry identified 108 interacting partners for MOSPD2 (Figure 5A), 7 of which also interacted with VAPA and VAPB. The partners either had a conventional FFAT motif (e.g., the Golgi protein STARD11 and the endosomal protein OSBPL1 (ORP1L)) or an FFAT-like motif (e.g., the endosomal proteins STARD3, STARD3NL and the mitochondrial protein PTPIP51). Di Mattia and colleagues [52] showed that the FFAT-like motifs that interact with MOSPD2 are phospho-FFAT motifs (described in Section 3.2), which depend on phosphorylation to mediate interaction. Endogenous MOSPD2 localizes to the ER, while the overexpression of its interacting partners such as STARD11, OSBPL1, STARD3, STARD3NL and PTPIP51 led to a recruitment of MOSPD2 to the contact sites of the ER and the Golgi, endosomes and mitochondria, respectively [21]. Although there is an overlap between the interactome of MOSPD2 and VAPA/VAPB (Figure 5B), the majority of the proteins identified are unique interactors of MOSPD2.

It is unclear how the balance of these individual VAP proteins is regulated at the MCSs of the ER. Their differential expression level is presumably a contributing factor. It was shown that in HeLa cells, VAPA and VAPB are 200- and 7-fold more abundant than MOSPD2, respectively [21]. Furthermore, the N-terminal CRAL-TRIO domain of MOSPD2 (Section 2.2), which has been associated with lipid transport in 28 human proteins [78], may affect binding to interaction partners.

### 4.4. Interactome of MOSPD1 and MOSPD3

Two other MSP domain-containing proteins encoded by the human genome are MOSPD1 and MOSPD3. Cabukusta and colleagues [22] also performed an extensive proteomic study using BioID followed by mass spectrometry to identify proteins interacting with MOSPD1 and MOSPD3 (Figure 5B). By assigning an FFAT score to the identified proteins they observed that MOSPD1 and MOSPD3 interact with proteins of higher FFAT score in comparison with VAPA, VAPB and MOSPD2. The well characterized interaction partners of VAPA and VAPB such as OSBP, STARD11, ORP1L and STARD3 with a lower FFAT score also showed an interaction with MOSPD2. Nevertheless, the selectivity towards all these proteins varied between VAPA/VAPB and MOSPD2. All the five VAP proteins interacted with PTPIP51 [22]. Furthermore, MOSPD1 and MOSPD3 do not interact with proteins containing conventional FFAT-, but instead with FFNT-motifs (Section 3.3). Among the interacting proteins identified, CEP85 (centrosomal protein 85), ANKLE2 (ankyrin repeat and LEM domain-containing 2), ENTR1 (endosome-associated-trafficking regulator 1) and the inner nuclear membrane protein emerin were specific to MOSPD1/3. Using proximity ligation assays, it was shown that the interaction between MOSPD3 and emerin occurs predominantly at the ER [22]. MOSPD1 and MOSPD3 can also form MCSs between the ER and mitochondria by their association with PTPIP51.

In summary, the VAP proteins can form distinct tethering complexes at the ER. VAPA-VAPB-MOSPD2 prefer FFAT motifs in their binding partners, whereas MOSPD1-MOSPD3 favor FFNT motifs. In addition, the ability of VAPs to form homomeric and heteromeric complexes via their transmembrane domains and/or their MSP domains favors this segregation and motif preferences [14,22,44].

## 5. Functional Significance of VAP Interactions

In light of their extensive interaction repertoire, is not surprising that VAP proteins have been implicated in the regulation of various cellular processes including membrane trafficking [17,89], lipid transport [71], calcium homeostasis [34] and the unfolded protein response [90,91]. However, their best described function is the regulation of lipid transport. Lipids synthesized in the ER are transported to their destination organelle by lipid transfer proteins, which interact with VAPs. For transferring ceramide between the ER and the Golgi, CERT (STARD11) interacts with VAPs (via its FFAT motif) of the ER membrane, extracts ceramide from it (via its START domain) and transfers it to phosphatidylinositol-4-phosphate of the trans-Golgi membrane (via its PH domain) [71]. Nir2 interacts with VAPA/VAPB and transfers phosphatidylinositol from the ER to the plasma membrane and also delivers phosphatidic acid to the ER [92]. Hyperphosphorylation of oxysterol binding protein 3 (ORP3) regulates its interaction with VAPA at the ER–plasma membrane contact sites and modulates its binding to phosphoinositides [93]. The VAPA/ORP3 interaction also promotes entry of late endosomes into the nucleoplasmic reticulum [94], and ORP3 has been reported to rescue the phenotype caused by ALS-linked VAPB P56S [95]. VAP proteins have also been implicated in regulation of microtubule organization. The interaction with Nir proteins (Nir1, Nir2 and Nir3) bridges VAPB to microtubules and regulates the ER structure [31]. While Nir2/VAPB interactions facilitate the formation of stacked ER membrane arrays, Nir3/VAPB interactions cause remodeling of the ER and microtubule bundling along the ER membranes. The Nir1/VAPB interaction, by contrast, has no effect on ER structure [31].

In contrast to VAPA and VAPB, the interaction studies for MOSPD proteins are very recent. MOSPD1 and MOSPD3 form contact sites with mitochondria by interacting with RMDN3. MOSPD3 depletion also delayed nuclear envelope reformation after mitosis [22]. Overall, a better understanding of the VAP proteins and their ability to interact with multiple partners would provide valuable insights into cellular functions.

## 6. Conclusions

**Figure 6:**
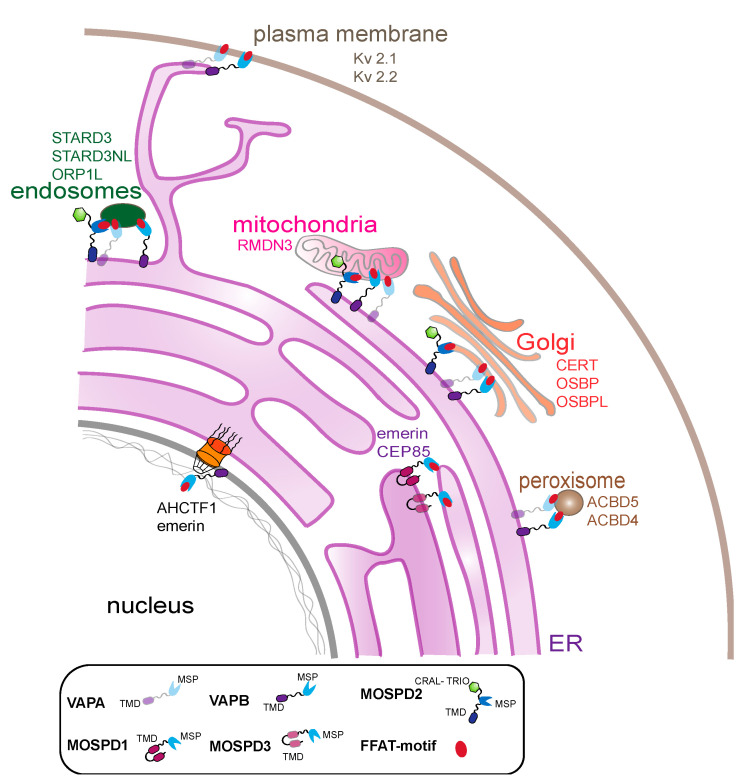
Proteins of the VAP family connect the ER and other organelles. Schematic representation of the interaction of all the family members with FFAT-motif at MCSs of the ER and endosomes, mitochondria, the plasma membrane, the Golgi apparatus, peroxisomes and the nuclear envelope. VAP proteins and a few examples of interacting proteins (FFAT-motif containing proteins) at the MCSs are depicted close to the organelles.

VAP proteins play an important role in tethering proteins in MCSs between the ER and other organelles (Figure 6). The interaction repertoire of VAPA, VAPB, MOSPD1, MOSPD2 and MOSPD3 has been expanding in the course of recent years [21,25,43,52,58,59]. The identification of FFAT-like motifs (phospho-FFAT, FFNT-motif) in addition to the conventional FFAT sequences substantially increased the number of interacting partners. Additionally, VAPs were shown to form two types of tethering complexes in the ER to mediate recruitment of proteins from different compartments, thereby expanding the interaction network at the MCSs. The different approaches used for analysis of the interactome markedly improved the mapping of distinct partners for each VAP protein. The traditional methods of affinity purification allow the identification of stable interaction partners, but transient interactors may be lost, depending on the buffer conditions. Proximity labelling methods using BioID and APEX, on the other hand, identify the spatial proteome within membrane boundaries. We modified the method to identify specific interactome of a pool of VAPB localized to the INM. However, the functional significance of these interactions remains to be investigated for most of the proteins. Since most of the studies are performed under conditions of VAP overexpression, chances are that some of the interactors are overexpression-induced artefacts. Hence, a proper validation of the interactome of the VAP proteins is necessary, and more work is required to understand the specific function of each of the VAP proteins.
